# Data quality and practical challenges of thyroid volume assessment by ultrasound under field conditions - observer errors may affect prevalence estimates of goitre

**DOI:** 10.1186/1475-2891-9-66

**Published:** 2010-12-14

**Authors:** Sigrun Henjum, Tor A Strand, Liv E Torheim, Arne Oshaug, Christine L Parr

**Affiliations:** 1 Akershus University College, P.O. Box 423, N-2001 Lillestrøm, Norway; 2 Centre for International Health, University of Bergen, Norway; 3 Medical Microbiology, Department of Laboratory Medicine, Innlandet Hospital Trust, Lillehammer, Norway; 4 Fafo Institute for Applied International Studies, Norway; 5 Institute of Basic Medical Sciences, Department of Biostatistics, University of Oslo, Norway

## Abstract

**Background:**

The ultrasonographic estimation of thyroid size has been advocated as being more precise than palpation to diagnose goitre. However, ultrasound also requires technical proficiency. This study was conducted among Saharawi refugees, where goitre is highly prevalent. The objectives were to assess the overall data quality of ultrasound measurements of thyroid volume (Tvol), including the intra- and inter-observer agreement, under field conditions, and to describe some of the practical challenges encountered.

**Methods:**

In 2007 a cross-sectional study of 419 children (6-14 years old) and 405 women (15-45 years old) was performed on a population of Saharawi refugees with prevalent goitre, who reside in the Algerian desert. Tvol was measured by two trained fieldworkers using portable ultrasound equipment (examiner 1 measured 406 individuals, and examiner 2, 418 individuals). Intra- and inter-observer agreement was estimated in 12 children selected from the study population but not part of the main study. In the main study, an observer error was found in one examiner whose ultrasound images were corrected by linear regression after printing and remeasuring a sample of 272 images.

**Results:**

The intra-observer agreement in Tvol was higher in examiner 1, with an intraclass correlation coefficient (ICC) of 0.97 (95% CI: 0.91, 0.99) compared to 0.86 (95% CI: 0.60, 0.96) in examiner 2. The ICC for inter-observer agreement in Tvol was 0.38 (95% CI: -0.20, 0.77). Linear regression coefficients indicated a significant scaling bias in the original measurements of the AP and ML diameter and a systematic underestimation of Tvol (a product of AP, ML, CC and a constant). The agreement between re-measured and original Tvol measured by ICC (95% CI) was 0.76 (0.71, 0.81). The agreement between re-measured and corrected Tvol measured by ICC (95% CI) was 0.97 (0.96, 0.97).

**Conclusions:**

An important challenge when using ultrasound to assess thyroid volume under field conditions is to recruit and train qualified personnel to perform the measurements. Methodological studies are important to assess data quality and can facilitate statistical corrections and improved estimates.

## Background

Accurate estimation of thyroid size is important for the evaluation and management of goitre and other thyroid disorders. Ultrasound is commonly used in epidemiologic studies [1-3], as a quick, safe, non-invasive technique to estimate thyroid volume (Tvol) [4,5]. Portable equipment makes ultrasound feasible even in remote areas [4,5] where goitre may be prevalent. Ultrasound has been advocated as being more precise than palpation in diagnosing goitre, but the interpretation of ultrasound scans is also somewhat subjective [5]. Small differences in ultrasound technique may introduce substantial errors into measurements of thyroid volume (Tvol), and the inter-observer variation can be high, even among experienced examiners [6]. Few studies have assessed the accuracy and variability of ultrasonic measurements of Tvol [6-8], and the data are limited to clinical settings. One challenge when using ultrasound in remote areas is to recruit qualified radiologists or to train personnel to perform the measurements [9].

Previous studies among Saharawi refugees residing in refugee camps near Tindouf in the Algerian desert have revealed a high prevalence of goitre, which is probably caused by iodine excess rather than iodine deficiency [10-13]. As part of a larger study of goitre among Saharawi refugees, the present methodological sub-study has two objectives: to assess the overall data quality of ultrasound measurements of Tvol, including the intra- and inter-observer agreement, under field conditions, and to describe some of the practical challenges encountered.

## Methods

### Population and data collection

A cross-sectional study was undertaken in January and February 2007 in four Saharawi refugee camps near Tindouf in the Algerian desert. The total population was estimated at approximately 165,000 persons. The target population was limited to children (6-14 years old) and women (15-45 years old). The sample size calculation was based on an estimated goitre prevalence of 50%, an absolute precision of ± 5% and a 95% confidence interval. This corresponded to approximately 400 children and 400 women, as determined with EpiInfo Statcalc; version 6.04b [14].

Each of the four refugee camps was organized into six administrative zones called "*dairas*", and it was assumed that each daira (24 in total) had approximately the same number of inhabitants. To achieve a total sample size of 800 individuals, about 34 participants (17 children and 17 women) were included from each daira from randomly selected households. The average household had 8 individuals in the target age groups, so about four households per daira were needed to reach the required number. All eligible children and women in each selected household were included. The total study sample included 421 children and 405 women from 92 households. The response rate was 96%, and the main reason for not participating was absence on the day of visit.

Ethical approval for the study was given by the Norwegian Regional Committee for Medical Research Ethics and by the Saharawi health authorities. Informed written consent was obtained from the chief medical officers in the camps. Informed oral consent was given by the women and the parents of the children included in the study. It was emphasised that refusal to participate in the study would have no negative effects on their entitlement to food aid or other services.

### Assessing thyroid volume

Tvol was assessed by two trained health workers (examiner 1 and 2): a trained nurse who completed a short course in ultrasound measurements at the Txagorritxu hospital in Vitoria-Gasteiz in Spain prior to the study and a local doctor who was trained by the nurse at the study site. Examiner 1 measured 406 individuals, and examiner 2, 418 individuals. A portable ultrasound (Sonosite Titan) equipped with a 38 mm 5-10 MHz linear transducer was used for the thyroid measurements. Subjects were examined in a supine position with extended cervical spine. Maximum perpendicular depth (anteroposterior, AP diameter) and width (mediolateral, ML diameter) were measured with electronic callipers on a transverse image of the largest diameter. The maximum lobe length (craniocaudal, CC diameter) was measured on a longitudinal image. Two images on a dual screen were used if the lobe extended beyond the 38 mm transducer measurement width. The transducer was kept perpendicular to the skin. Nodules and/or cystic areas were included in the volume determination. Thyroid volume was estimated according to the method of Brunn et al. [15]. By regarding the two lobes without the isthmus as corrected rotation ellipsoids, the volume of the thyroid gland was calculated by adding the volume of the right and left lobe, each calculated as follows: Tvol _lobe _= AP diameter × ML diameter × CC diameter × 0.479. A thyroid enlargement in a child was regarded as a goitre if the thyroid volume was above the 97th percentile of the age and sex-specific international reference values for thyroid volumes in an iodine-replete population [5]. Thyroid enlargement was defined as a Tvol exceeding 18 ml for women, which corresponds to mean + 3SD in iodine sufficient populations [16]. Height and weight were measured by standard anthropometric techniques [17]. Body weight was measured using a UNICEF electronic SECA 890 (Hamburg, Germany) to the nearest 0.1 kg. Height was measured to the nearest 0.1 cm using a portable stadiometer. Spot urine samples were aliquoted and stored at 5°C until analysis. Water samples were collected in every household. To determine the urinary iodine concentration (UIC) and the iodine concentration in the household drinking water, samples were analysed according to the Sandell-Kolthoff reaction [18].

### Intra- and inter-observer agreement

To assess intra- and inter-observer agreement of the ultrasonic measurements, a methodological sub-study was conducted on 12 children (7 to 14 years old), who were selected from the study population, but were not part of the main study. Each field worker measured the 12 children twice using the same apparatus. Thus, a total of 48 Tvol measurements were taken. To minimize any influence of the fieldworkers' memory, the time between measurements of the same individual was increased by measuring all subjects once in consecutive order from 1 to 12 before repeating the procedure. Since each Tvol measurement involved the evaluation of up to six images per person (an image set) - 4 transverse images (two diameters on each lobe, right and left sides) and up to 2 longitudinal images - it is unlikely that the fieldworkers would remember the specific measurements of any individual.

### Clinical evaluation of ultrasound images

A sample of 56 images (28 from each examiner) of all diameters was sent to an external ultrasound expert at the Txagorritxu hospital in Vitoria-Gasteiz, Spain, who inspected the images visually. According to the expert evaluation, the ultrasound measurements of examiner 1 were satisfactory. The AP diameters measured by examiner 2 were consistently too short, nor were they perpendicular to the ML diameter. The CC diameter measurements were also evaluated, but not commented upon in the expert evaluation.

### Validation study and re-measurement of ultrasound images

Based on the study of intra- and inter-observer agreement and the clinical evaluation, the decision was made to print and re-measure the images taken by examiner 2. The images of examiner 1 had been found to be satisfactory, so examiner 1 also performed the new measurements. Only 272 of the 418 image sets for examiner 2 had been stored and could be retrieved. New measurements of the AP and ML diameters were taken. The original CC diameters were retained because the quality of the stored images was generally insufficient for taking new measurements.

### Statistical methods

Data on Tvol, UIC, and iodine in drinking water did not adhere to a Gaussian distribution. Descriptive statistics were therefore reported as the median value with percentiles (P_25 _and P_75_). and the difference in Tvol between the samples of the two examiners was tested using the Mann-Whitney test. The proportion of goitre in women and children was compared with a Chi-square test. All P values were two-sided, and a 5 percent significance level was used.

The intra- and inter-observer agreement in the measurements of the thyroid diameters and thyroid volume were presented as intraclass correlation coefficients (ICC) based on a one-way random effects model for single measurements taking the absolute agreement between the measurements into account [19]. The ICCs express proportions of variance, and when a high proportion of the total variance is accounted for by intra- or inter-observer variability, the corresponding ICC and agreement in the measurements will be low. For comparability with other studies, the inter-observer variability was also calculated as the absolute value of the difference between the measurements of examiner 1 and examiner 2 expressed as a percentage of the mean of the two measurements for each individual [7]. This percentage was presented as the sample mean value (%) and standard deviation (SD). Only the first measurement of each examiner was included in the calculation of inter-observer variability.

The measurements of examiner 2 were corrected statistically by regressing the re-measured values (considered to be the "true" value) for the AP and ML diameters on the original measurements using linear regression for the sub-sample of available images (n = 272). The corrected values were then predicted with the regression equations and used to re-calculate Tvol (retaining the original CC diameter, which could not be re-measured) for the 418 individuals measured by examiner 2. A regression equation for Tvol was also used to directly predict the corrected value from the original value. The agreement between the original and re-measured Tvol values and between the re-measured and statistically corrected Tvol values was summarized using ICC. SPSS version 14.0 (SPSS Inc., Chicago) was used for the analyses.

## Results

As shown in Table 1, the background characteristics were similar for the study participants assessed by the two examiners.

**Table 1:** Selected background characteristics of children (n = 419) and women (n = 405) in 92 households, stratified for examiners 1 and 2.

	Examiner 1 (n = 406)	Examiner 2 (n = 418)
**Children, n (%)**	209 (51.5)	210 (50.2)
Age in years, mean (SD)	10.2 (2.6)	9.7 (2.6)
Urinary iodine (μg/L), median (P_25_-P_75_)	597 (366-917)	536 (346-885)
**Women, n (%)**	197 (48.5)	208 (49.8)
Age in years, mean (SD)	29.2 (8.5)	29.0 (8.5)
BMI (kg/m^2^), mean (SD)	25.0 (4.6)	25.2 (6.5)
Urinary iodine (μg/L), median (P_25_-P_75_)	476 (294-772)	459 (301-705)
**Households, n (%)**	46 (50)	46 (50)
Iodine in drinking water (μg/L), median (P_25_P_75_)	112 (83-301)	103 (72-297)

The sub-study of intra- and inter-observer agreement is presented in Tables 2 and 3, respectively. The intra-observer agreement in Tvol was higher in examiner 1 with an ICC (95% CI) of 0.97 (0.91, 0.99) compared to 0.86 (0.60, 0.96) in examiner 2 (Table 2). The ICC (95% CI) for inter-observer agreement in Tvol was 0.38 (-0.20, 0.77) (Table 3). The mean (SD) inter-observer variability for Tvol (calculated as the absolute difference between examiners 1 and 2 divided by the mean Tvol) was 36 (14)%.

**Table 2:** Intra-observer agreement of thyroid gland diameters and thyroid volume (Tvol) measured by portable ultrasound in children aged 6-14 years (n = 12).

	Right lobe			Left lobe			Tvol
	**ML**	**AP**	**CC**	**ML**	**AP**	**CC**	
**Examiner 1**							
ICC*	0.67	0.88	0.89	0.85	0.78	0.74	0.97
**(95% CI)**	(0.20, 0.89)	(0.65, 0.96)	(0.67, 0.97)	(0.59, 0.96)	(0.42, 0.93)	(0.33, 0.92)	(0.91, 0.99)
**Examiner 2**							
ICC*	0.67	0.55	0.28	0.54	0.63	0.72	0.86
**(95% CI)**	(0.21, 0.89)	(0.02, 0.85)	(-0.30, 0.72)	(0.02, 0.84)	(0.15, 0.88)	(0.30, 0.91)	(0.60, 0.96)

*ICC intraclass correlation coefficient, one-way random model (measure of agreement)

**Table 3:** Inter-observer agreement of thyroid gland diameters and thyroid volume (Tvol) measured by portable ultrasound in children aged 6-14 years (n = 12), in examiners 1 and 2.

	Right lobe			Left lobe			Tvol
	**ML**	**AP**	**CC**	**ML**	**AP**	**CC**	
ICC*	0.48	-0.04	0.28	0.59	0.50	-0.19	0.38
**(95% CI)**	(-0.08, 0.81)	(-0.77, 0.19)	(-0.31, 0.72)	(0.08, 0.86)	(-0.05, 0.82)	(-0.66, 0.40)	(-0.20, 0.77)

*ICC intraclass correlation coefficient, one-way random model (measure of agreement)

The linear regression coefficients for predicting the corrected measurements for the AP and ML diameters and for Tvol directly are presented in Table 4. The coefficients indicate a significant scaling bias (given by β_1_) in the original measurements of the AP and ML diameters (both lobes) and calculated Tvol. A small, but significant positive additive error (given by β_0_) was found for the ML diameter (both lobes) and the AP diameter (right lobe only), but not for Tvol. Tvol (a product of AP, ML, CC and a constant), was systematically underestimated. The coefficients for directly predicting the corrected measurement for Tvol were used in the rest of the article.

**Table 4:** Linear regression equation coefficients for directly predicting corrected measurement^a^ of the anteroposterior (AP) and mediolateral (ML) diameters, and thyroid volume (Tvol) as a function of the original measurements (n = 272).

	**Beta**_**1 **_**(95% CI)**	**Beta**_**0 **_**(95% CI)**
**Right lobe**		
**ML**	0.88 (0.83, 0.94)	0.35 (0.27, 0.43)
**AP**	1.11 (1.03, 1.20)	0.18 (0.09, 0.26)
**Left lobe**		
**ML**	0.87 (0.79, 0.94)	0.45 (0.33, 0.57)
**AP**	1.18 (1.10, 1.27)	0.01 (-0.07, 0.09)

**Tvol**	1.42 (1.37, 1.46)	0.01 (-0.25, 0.27)

^a^Corrected = β_1* _original + β_0_

Before the correction, median Tvol was higher in examiner 1 compared to examiner 2 for both children (5.7 versus 3.3 ml, respectively p < 0.001) and women (10.3 versus 5.8 ml respectively p < 0.001) (Table 5). After the correction, the median Tvol (P_25_-P_75_) of examiner 2 increased from 3.3 ml (2.3-4.2) to 4.6 ml (3.2-6.0) in children, and from 5.8 ml (4.6-7.6) to 8.3 ml (6.6-10.8) in women. The corresponding prevalence of goitre increased from 11% to 44% in children and from 1% to 3% in women. In children, the difference in prevalence between examiner 1 and 2 remained significant (p < 0.001).

**Table 5:** Thyroid volume (Tvol) and goitre prevalence in children (n = 419) and women (n = 405), stratified for examiners 1 and 2, before and after the correction of examiner 2's Tvol.

	Examiner 1 (n = 406)	Examiner 2 (n = 418)		P-value
	
		Before correction	After correction	
**Children, (n)**	209	210	210	-
Tvol (mL), median (P_25_-P_75_)	5.7 (4.3-7.6)	3.3 (2.3-4.2)	4.6 (3.2-6.0)	<0.001^c^
Goitre^a ^n (%)	112 (69)	18 (11)	75 (44)	<0.001^d^
**Women, (n)**	197	208	208	-
Tvol (mL), median (P_25_-P_75_)	10.3 (8.6-12.9)	5.8 (4.6-7.6)	8.3 (6.6-10.8)	<0.001^c^
Goitre^b ^n (%)	11 (6)	2 (1)	7 (3)	0.01/0.34^d^

^a^International reference values for Tvol for age [5]

^b^Thyroid enlargement was defined as a Tvol exceeding 18 ml for women [16]

^c^Mann-Whitney test (examiner 1 and examiner 2, same p-value before and after correction)

^d^Chi-square test (examiner 1 and examiner 2, before and after correction)

Among the participants assessed by examiner 2, there was little difference in the original median (P_25_-P_75_) Tvol among the 272 participants (65%) with images that could be retrieved and re-measured compared to Tvol in the 146 participants (35%) with non-retrievable images: 4.2 (3.0, 6.0) and 4.9 (3.2, 6.5), respectively (p = 0.06)

The agreement between the re-measured and original Tvol measured by ICC (95% CI) was 0.76 (0.71, 0.81). The agreement between the re-measured and corrected Tvol measured by ICC (95% CI) was 0.97 (0.96, 0.97).

## Discussion

In this methodological sub-study of thyroid volume measurements by ultrasound under field conditions, low intra-observer agreement for one of the examiners was found. This contributed to the low inter-observer agreement and the detection of a systematic observer error in estimated Tvol, which was partly corrected.

### Correction of the measurement error and the effect on the prevalence estimates

The methodological sub-study and clinical evaluation of the ultrasound images lead to the detection of observer error in the ultrasound measurements of examiner 2. There were no differences in the selected background characteristics of the two study samples that could otherwise explain the difference in Tvol. The two examiners visited different households, but operated in the same neighbourhoods, in the same camps. Therefore, it was unexpected to find such a difference in Tvol. The development and application of a correction factor seemed appropriate for several reasons. First, the observer error in Tvol was clearly systematic. Second, a large proportion of the images of examiner 2 were re-measured (65%), and little difference was found in the original Tvol between participants with retrievable and non-retrievable images, indicating that the estimated correction factor was probably representative for the whole sample of examiner 2. Finally, the agreement between the statistically corrected Tvol and the re-measured Tvol was very high (ICC = 0.97).

The application of a correction factor for the systematic error in this study sharply reduced the difference in Tvol and goitre prevalence between the two examiners, but a difference still remained. One possible explanation is residual measurement error in the CC diameter, which could not be re-measured and corrected because the quality of the stored images was suboptimal. Examiner 2 used one single scan to measure the CC diameter more often than examiner 1, instead of taking time to merge two images. This may indicate that too little time was spent searching for optimal images, or there may have been time pressure. Another explanation is that the Tvol measurements of examiner 1 were biased. However, the external ultrasound expert reported that the Tvol measurements of examiner 1 were of satisfying quality. Finally, there may have been real differences between the two study samples.

### Challenges in thyroid volume assessment under field conditions

Goitre is a problem in several low-income countries, where ultrasound measurements may be difficult to conduct. In this study, we faced several problems ranging from identification of trained personnel, language barriers and visa issues to electricity problems, among others. We believe that these problems are not unique to our setting. An important challenge when using ultrasound in remote areas is to recruit and train qualified personnel to perform the measurements. Many low-income countries have a shortage of radiologists [9]. Ultrasound is totally operator dependent; the equipment may be easy to operate, but the images are equally easy to misinterpret. Training and experience are necessary in order to capture high quality images and correctly measure the various diameters of the thyroid gland that are then used to calculate thyroid volume (Tvol) [5]. Ultrasound has no value as a major diagnostic tool if the imager is inadequately trained or inexperienced [9]. In our study, examiner 1 was more experienced in ultrasound measurements than examiner 2. Examiner 2 was denied a visa to Spain where the ultrasound training was performed (Txagorritxu hospital in Vitoria-Gasteiz, Spain). Thus, examiner 1 received formal training whereas examiner 2 had to be trained by examiner 1. The examiners in our study used the same ultrasound equipment model. However, it was discovered that examiner 2 had an ultrasound machine with a battery that had to be recharged more often than the battery in examiner 1 machine. Identifying the maximum diameters takes time. Thus, the fact that examiner 2's CC diameters were more often on one single scan can be attributed to time pressure to complete the measurements before the equipment had to be recharged. When the battery needed charging, examiner 2 had to continue the Tvol measurements in the back of a car, a less than optimal situation for performing accurate ultrasound measurements.

### Intra- and inter-observer agreement

In our study the intra-observer agreement in examiner 1 was higher than in examiner 2. This indicates that the Tvol measurements of examiner 2 were less consistent. The inter-observer agreement was lower than the intra-observer agreement, which was expected and also in accordance with previous studies [6-8].

Inter-observer variation for Tvol measurements in schoolchildren has been reported to be in the range of 3-13% [7,20-22]. In a study by Zimmermann et al. [6], where experienced examiners performed the Tvol measurements, a higher inter-observer variation (26%) was found. This result was partly attributed to a large systematic bias in one of the examiners and a correction factor was developed [6]. When applied to the data, the discrepancy between the examiners was sharply reduced. In our study trained fieldworkers measured Tvol, so a slightly higher inter-observer variability of 36% may be expected compared to the 26% found in Zimmermann's study. In 2007 when the present study was undertaken, there was no published standard for ultrasound assessment of Tvol. A defined and detailed description of the ultrasound technique at the time would have been a helpful tool and could possibly have reduced measurement error in the data. A standard has now been published by WHO [4].

## Conclusion

An important challenge when using ultrasound to assess thyroid volume under field conditions is to recruit and train qualified personnel to perform the measurements. Methodological studies are important to assess data quality and can facilitate statistical corrections and improved estimates.

## Competing interests

The authors declare that they have no competing interests.

## Authors' contributions

SH played an active role in the design and planning of the study, supervised the data collection, carried out the statistical analyses, prepared tables and figures, and drafted the manuscript, taking into account comments from all co-authors. LET, TAS and AO were responsible for planning and designing the current study. CLP and TAS contributed to the statistical analysis and data interpretation. All authors read and approved the final manuscript.
